# Improving Astrometric Precision with MLP-Driven Super-Resolution of Star Maps

**DOI:** 10.3390/s26061769

**Published:** 2026-03-11

**Authors:** Yi Lu, Xiping Xu, Juncen Yan, Ning Zhang, Yaowen Lv

**Affiliations:** National Demonstration Center for Experimental Opto-Electronic Engineering Education, School of Opto-Electronic Engineering, Changchun University of Science and Technology, Changchun 130022, China; yjc12342024@163.com (J.Y.); zhangning@cust.edu.cn (N.Z.); lyw@cust.edu.cn (Y.L.)

**Keywords:** dynamic star simulator, multi-layer perceptron, sub-pixel, super-resolution correction, aberration field modeling

## Abstract

Aiming at the star centroid positioning error in dynamic star simulators, a super-resolution star map correction method is proposed based on a multi-layer perceptron (MLP). A complete technical chain of “system calibration–aberration field modeling–network correction” is constructed to establish a data-driven end-to-end framework for unified modeling and compensation of optical aberrations, assembly deviations, and device discreteness. Experimental results show that the proposed method achieves sub-pixel accuracy: the maximum star centroid and inter-star angular distance errors are reduced by 22.9% and 37.5% on average, respectively, which is significantly superior to traditional methods. This work provides a reliable technical approach for high-precision star map display and star sensor ground calibration, with clear engineering application value.

## 1. Introduction

The dynamic star simulator is an essential facility for the ground testing and performance calibration of star trackers [1]. The accuracy with which it simulates stellar positions and spacecraft attitude fundamentally determines the navigation precision and operational reliability of star trackers in orbit [2]. Consequently, this technology is of significance in various fields, including autonomous spacecraft navigation, deep-space exploration, high-precision astronomical observation, and space situational awareness [3]. As performance expectations for navigation systems in aerospace missions escalate, star simulators face increasingly stringent requirements in stellar positional accuracy, dynamic range, and system-wide stability. However, inherent optical aberrations, manufacturing and alignment tolerances in mechanical components, and intrinsic pixel structures with nonlinear responses in display devices collectively impose fundamental limitations on sustainably improving system performance through hardware optimization alone [4]. Consequently, the establishment of error models combined with the implementation of corresponding compensation techniques has evolved into an essential technological strategy for mitigating hardware constraints and enabling high-fidelity star field simulation [5].

Focusing on the core objective of high-precision correction, many studies have been conducted on techniques ranging from local compensation to system modeling. In [6], a multi-star independent focal-length correction method was adopted, which effectively enhanced the starlight emission accuracy. However, this study failed to perform an in-depth analysis of their underlying causes or implement corresponding compensatory measures, although it identified the limitations of the single focal-length correction model. In [7], a star point position correction method based on polar coordinates was proposed, achieving a starlight emission accuracy of better than ±22 arcseconds within a 22° field of view. Despite notable progress in the accuracy correction of star simulators for specific scenarios, a comprehensive correction model for star simulators remains underdeveloped. In 2022, ref. [8] employed sub-pixel image display technology to allocate the energy of star points, thereby ensuring that the energy distribution conforms to a two-dimensional Gaussian distribution; this method significantly improved the star point simulation accuracy for specific systems. In [9], a star point extraction error correction model based on wave aberration was established by analyzing the aberrations of the optical system, achieving a starlight emission accuracy exceeding 10 arcseconds. Nevertheless, the study did not perform an analysis or calculation of the effects of coma aberration, chromatic difference of magnification, and other aberrations on star point positions. In [10], a star point position correction method that accounts for the effects of aberrations such as distortion, coma aberration, and field curvature was proposed. After correction, the maximum error in starlight emission accuracy was reduced to 10.75 arcseconds; however, influential factors such as manufacturing and alignment errors were not considered. In the same year, ref. [11] proposed a high-precision correction method with zonal refinement. On the basis of dividing the field of view into four quadrants, high-error regions were further subdivided, resulting in a maximum single-star position error of less than 18 arcseconds after correction. In 2025, ref. [12] proposed a starlight emission accuracy compensation method based on point-by-point focal length, establishing a differentiated correction model tailored to the characteristics of star points in different fields of view. Compared with traditional unified focal-length correction methods and zonal correction methods, this approach further improves the overall correction accuracy of the system while reducing the number of iterations.

As can be seen from the above research work, existing studies have made significant progress in improving the simulation accuracy of star spot positions by introducing methods such as independent focal length correction for multiple star spots, polar coordinate transformation, sub-pixel energy distribution, wave aberration modeling, and point-by-point focal length compensation. However, these methods mostly focus on local compensation for specific error sources or rely on simplified model assumptions. They have not yet established a global high-precision correction model that can uniformly characterize the coupled effects of multiple factors, such as aberrations, assembly errors, and device characteristics. Thus, significant bottlenecks still exist in further improving the accuracy and system versatility. To address the aforementioned issues, this paper proposes a star map super-resolution correction method based on a multi-layer perceptron (MLP) and constructs an end-to-end correction pipeline that maps ideal star maps to high-precision rendered outputs. This method circumvents the limitations of high model complexity and poor adaptability inherent in traditional zonal correction schemes, enabling high-precision full-field reconstruction of star spot positions. In doing so, it provides a practical and effective technical framework for the high-precision calibration of star map display systems while also offering a novel avenue for the development of next-generation high-precision star simulators.

## 2. Principles of Star Map Simulation and Correction

### 2.1. Principles of Star Map Simulation and Geometric Correction Model

**Figure 1:**
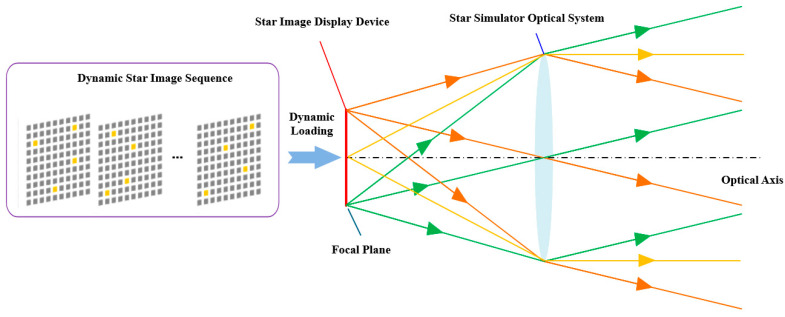
Principles of star map simulation for fixed stars.

The operating principle of the star simulator lies in precisely positioning the star map display device at the focal plane of the optical system while maintaining its perpendicularity to the optical axis [13]. By loading dynamic star map sequences onto the device, realistic simulations of celestial scenes observed under specific pointing directions within the celestial inertial coordinate system can be achieved [14]. As schematically illustrated in Figure 1, this constitutes the fundamental working mechanism of the system.

Drawing upon the analysis of the imaging principles and optical aberration characteristics of the star simulator, the distortion within the star simulator’s optical system typically exhibits a rotationally symmetric distribution about the center, provided that the system maintains high-quality imaging performance [15]. Such distortion induces a deviation of the actual chief ray from the ideal image point, consequently degrading the positional accuracy of the imaged star spots [16]. Furthermore, due to inherent uncertainties in mechanical structure design and system alignment processes, star simulators are prone to discrepancies such as principal point offset, image plane tilt, and focal length deviation [17]. All the aforementioned errors ultimately manifest as positional shifts of star spots on the display device, so it is necessary to carry on the compensation through adjustments to the display coordinates of the star spots [18].

Let the azimuth and elevation angles of the field-of-view (FOV) center point be denoted as $\alpha_{0}$ and $\beta_{0}$ (typically set to 0, respectively). Suppose that the azimuth and elevation angles of the star spot position A before correction are $\alpha_{1}$ and $\beta_{1}$, while those of the corrected star spot position B are $\alpha_{2}$ and $\beta_{2}$. Then, the angular distance between a single star and the FOV center for both points A and B can be uniformly expressed as

(1) $$\gamma_{i}=arccos[cos\beta_{0}cos\beta_{i}cos(\alpha_{0}-\alpha_{i})+sin\beta_{0}sin\beta_{i}]$$

By substituting the pre-correction and post-correction angular values separately, the angular distances of the single star before and after correction, denoted as $\gamma_{1}$ and $\gamma_{2}$, can be derived. Correspondingly, the angular correction value Δγ is expressed as

(2) $$\Delta \gamma =\gamma_{2}-\gamma_{1}$$

**Figure 2:**
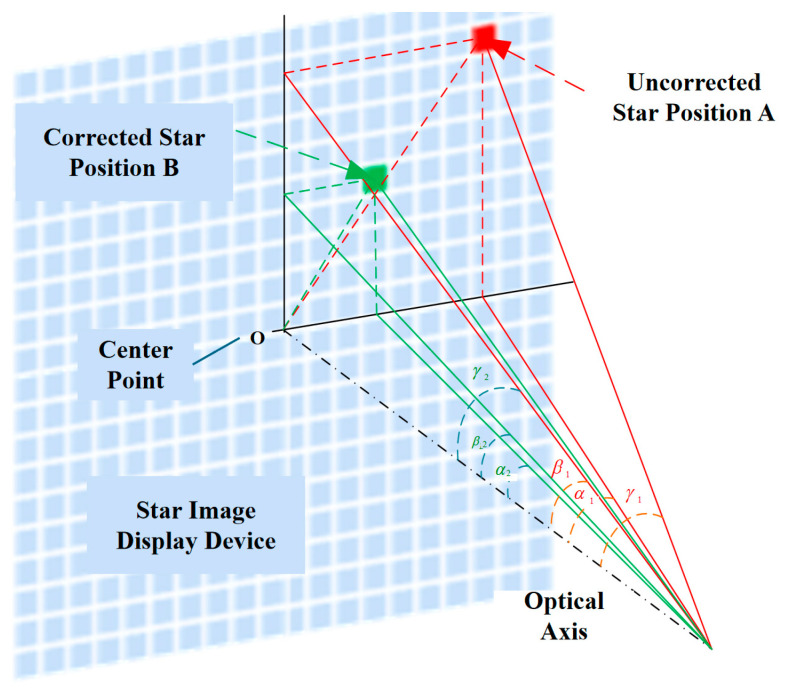
Geometric correction model for star maps.

Based on the aforementioned principles, a correction model for fixed star maps is established, as illustrated in Figure 2.

### 2.2. Principles of Super-Resolution Correction Based on Energy Regulation

**Figure 3:**
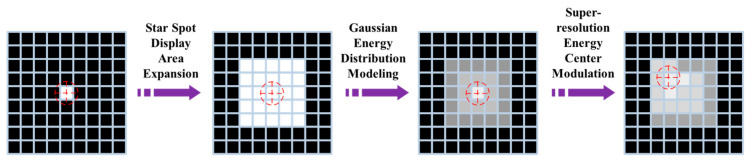
Principle of super-resolution correction based on energy modulation.

Constrained by the inherent discreteness of star map display devices, star spot coordinates must be quantized to integer pixel positions, thereby confining the accuracy of traditional geometric correction methods to their theoretical upper limits [19]. To overcome this limitation, a single-pixel star spot can be expanded into a display area composed of multiple pixels, thereby enhancing the correction accuracy [20]. Given that the point spread function (PSF) of the optical system allows the energy distribution profile of a star spot to be approximated by a two-dimensional Gaussian function, this distribution model can be leveraged to coordinate the brightness distribution of individual pixels within the display area via grayscale modulation, thereby achieving sub-pixel-level precision in the localization of the star spot’s energy center [21]. Accordingly, the principle underlying star map super-resolution reconstruction can be decomposed into three sequential steps: star spot display area expansion, Gaussian energy distribution modeling, and super-resolution center modulation. The principle of super-resolution correction based on energy regulation is schematically illustrated in Figure 3.

#### 2.2.1. Expansion of the Star Spot Display Region

The expansion of the star spot display region entails extending the star spot display unit from a single pixel to a rectangular region consisting of M×N pixels. The mathematical definition of this extended region R is given by

(3) R={(i,j)∣i∈[1,M],j∈[1,N]}

where i and j denote the coordinate indices of pixels within the region.

#### 2.2.2. Modeling of Gaussian Energy Distribution

Modeling of the Gaussian energy distribution aims to assign grayscale values to individual pixels within the expanded star spot display region in accordance with the probability density distribution of the two-dimensional Gaussian function, with its mathematical expression given by

(4) $$I(x,y)=I_{0}\cdot \exp\left(-\frac{{\left(x-x_{0}\right)}^{2}+{\left(y-y_{0}\right)}^{2}}{2\sigma^{2}}\right)$$

where I(x,y) denotes the light intensity at coordinate (x,y), $I_{0}$ is the peak light intensity at the star spot center, $\left(x_{0},y_{0}\right)$ represents the theoretical energy center coordinate of the star spot, and σ denotes the standard deviation of the Gaussian distribution, which dictates the dispersion level of the light spot, which physically represents the dispersion level of the star spot—a larger value indicates a more spread-out energy distribution, while a smaller value indicates energy more concentrated at the center.

#### 2.2.3. Modulation of the Super-Resolution Energy Center

Modulation of the super-resolution energy center enables the precise alignment of the star spot’s energy center with the target sub-pixel position $\left(x_{c},y_{c}\right)$ via the regulation of grayscale values for individual pixels within the display region, with the specific calculation formula expressed as follows:

(5) $$x_{c}=\frac{\sum_{i=1}^{M}\sum_{j=1}^{N}g_{ij}\cdot x_{ij}}{\sum_{i=1}^{M}\sum_{j=1}^{N}g_{ij}}\;y_{c}=\frac{\sum_{i=1}^{M}\sum_{j=1}^{N}g_{ij}\cdot y_{ij}}{\sum_{i=1}^{M}\sum_{j=1}^{N}g_{ij}}$$

where $g_{ij}$ denotes the grayscale value at the i-th row and j-th column, and $\left(x_{ij},y_{ij}\right)$ represents the central coordinate of this pixel.

To achieve high-precision sub-pixel localization, it is necessary to solve for the optimal grayscale value $\left\{g_{ij}\right\}$ of each pixel, such that the error between the energy center $\left(x_{c},y_{c}\right)$ of the entire star spot and the target sub-pixel position $\left(x_{t},y_{t}\right)$ is minimized. Accordingly, this problem can be formulated as a constrained optimization problem, expressed as

(6) $$\underset{\left\{g_{ij}\right\}}{\min}\left[{\left(x_{c}-x_{t}\right)}^{2}+{\left(y_{c}-y_{t}\right)}^{2}\right]$$

Constraints $0\leq g_{ij}\leq g_{\max}$ and $\sum g_{ij}=E$ must be satisfied simultaneously, where $g_{\max}$ denotes the grayscale value range constraint and E represents the total energy of the star spot (which is correlated with stellar magnitude). The optimization aims to precisely adjust the position of the energy center to achieve sub-pixel-level positioning.

To simplify computational complexity, the target grayscale distribution $\left\{g_{ij}\right\}$ can be designed to match the form of the Gaussian model. Taking the logarithm of both sides of the Gaussian distribution function yields

(7) $$\ln I(x,y)=\ln I_{0}-\frac{{\left(x-x_{0}\right)}^{2}+{\left(y-y_{0}\right)}^{2}}{2\sigma^{2}}$$

Let $z=\ln I\left(x,y\right)$, $a=\ln I_{0}$ and $b=-\frac{1}{2\sigma^{2}}$ be given, and the resulting linear relationship is expressed as

(8) $$z=a+b[{\left(x-x_{0}\right)}^{2}+{\left(y-y_{0}\right)}^{2}]$$

The parameters a and b can be derived by fitting the relationship between the grayscale values and spatial positions of individual pixels within the display region using the least-squares method, thereby determining the theoretical grayscale value $g_{ij}=e^{z_{ij}}$ corresponding to each pixel. The approach can effectively enhance the localization accuracy of the energy center based on the grayscale distribution, thereby meeting the requirements of high-precision star map simulation.

## 3. Star Map Correction Algorithm Based on Multi-Layer Perceptron

**Figure 4:**
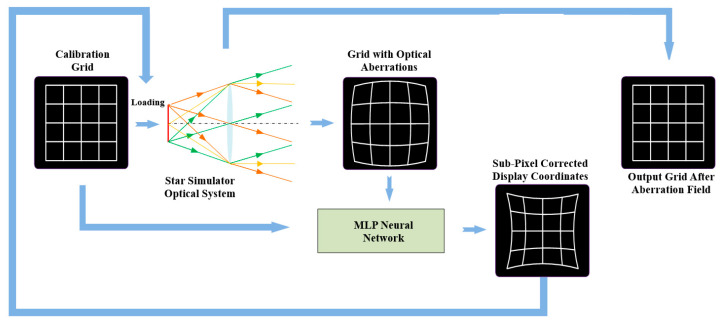
Workflow of the stellar star map correction algorithm based on MLP.

Based on the analysis presented in Section 2.1, all categories of systematic errors are ultimately manifested as positional deviations of star spots on the display device. To address this issue, this paper proposes an end-to-end correction scheme based on an MLP. This scheme first uniformly models the comprehensive errors induced by factors such as optical aberrations, mechanical assembly deviations, and device discreteness as a systematic aberration field and achieves its complete characterization using grid point calibration data. Subsequently, an MLP network is constructed to establish a nonlinear mapping from theoretical coordinates to super-resolution corrected coordinates based on deep learning. Finally, the trained network is employed to perform real-time inference on arbitrary input star maps, directly outputting the display coordinates corrected to sub-pixel-level precision. After propagating through the aberration field of the optical system, these coordinates can accurately reproduce the star spot distribution of the calibrated grid, thereby achieving a high-precision simulation of the real starry sky. This data-driven approach effectively overcomes the limitations of complex modeling and poor adaptability inherent in traditional zonal correction methods, providing a novel technical pathway for high-precision star map display and significantly enhancing the accuracy and efficiency of ground calibration for star sensors. The workflow of the star map correction algorithm based on the MLP is illustrated in Figure 4.

### 3.1. System Calibration and Aberration Field Construction

The system calibration stage aims to obtain the comprehensive aberration characteristics of the optical system via precise experimental measurements so as to provide input parameters for the subsequent MLP network.

Within the full field of view, uniformly distributed grid points of 21 × 21 are adopted as the calibration reference. A high-precision theodolite is used to measure the actual azimuth $\alpha_{m}$ and elevation angle $\beta_{m}$ of each grid point, while the ideal azimuth $\alpha_{t}$ and elevation angle $\beta_{t}$ are recorded. The theoretical planar coordinates $\left(u_{t},v_{t}\right)$ and measured planar coordinates $\left(u_{m},v_{m}\right)$ of the star points are obtained through coordinate transformation.

(9) $$\left(u,v\right)=\left(\tan\alpha ,\tan\beta \right)$$

On the basis of the aberration theory [20], the mathematical model of the aberration field, including radial distortion coefficients and decentering distortion coefficients, is established as follows:

(10) $$\left\{\begin{array}{l} u_{m}=u_{t}+u_{t}(k_{1}r_{t}^{2}+k_{2}r_{t}^{4}+k_{3}r_{t}^{6})+p_{1}(r_{t}^{2}+2u_{t}^{2})+2p_{2}u_{t}v_{t}\\ v_{m}=v_{t}+v_{t}(k_{1}r_{t}^{2}+k_{2}r_{t}^{4}+k_{3}r_{t}^{6})+p_{2}(r_{t}^{2}+2v_{t}^{2})+2p_{1}u_{t}v_{t} \end{array}\right.$$

where $r_{t}^{2}=u_{t}^{2}+v_{t}^{2}$ denotes the normalized radius; $k_{1}$, $k_{2}$, and $k_{3}$ denote the first-order, second-order, and third-order radial distortion coefficients of the optical system, respectively; and $p_{1}$ and $p_{2}$ denote the decentering distortion coefficients in the *x*- and *y*-directions of the optical system, respectively.

By applying the least-squares method, an overdetermined system of equations Ak=b is constructed, and the system aberration parameter vector $k={\left[k_{1},k_{2},k_{3},p_{1},p_{2}\right]}^{T}$ is solved as follows:

(11) $$k={\left(A^{T}A\right)}^{-1}A^{T}b$$

where A denotes the coefficient matrix and b denotes the observation vector.

The obtained aberration parameter vector is used as part of the network input for training the MLP to establish the mapping from theoretical coordinates to corrected coordinates.

### 3.2. Network Model Construction and Training

In this paper, the aberration parameters are selected as the input to the MLP network, rather than directly using the uncorrected centroid coordinates, mainly based on the following considerations. Traditional methods directly employ the centroid coordinates of discrete sampling points for zonal fitting, which suffer from problems such as a limited number of sampling points, unavoidable measurement deviations, and discontinuities at partition boundaries. The aberration parameters (radial distortion coefficients k_1_, k_2_, and k_3_ and decentering distortion coefficients p_1_ and p_2_) constitute a compact representation of the system aberration field obtained by least-squares fitting based on the entire calibration grid data, which can encapsulate the distortion law over the full field of view. By taking them as inputs, the MLP network can learn the continuous mapping function from theoretical coordinates to corrected coordinates and realize prediction for arbitrary input coordinates, thereby breaking through the limitations of traditional methods. The network model employs a general architecture composed of one input layer, three hidden layers, and one output layer.

During the network model construction and training stage, the objective is to train the MLP network to establish a nonlinear mapping from theoretical coordinates to corrected coordinates based on the aberration field parameters obtained from system calibration so as to provide model support for the real-time inference stage.

**Figure 5:**
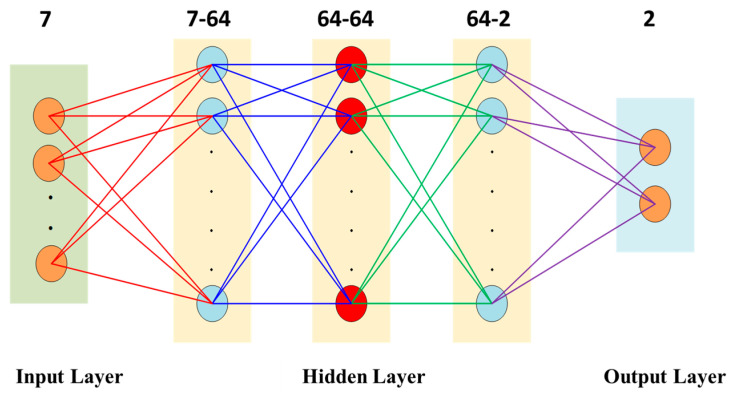
Workflow of the stellar star map correction algorithm based on a multi-layer perceptron (MLP).

The network adopts an overall architecture consisting of one input layer, three hidden layers, and one output layer. The input layer receives the grid-normalized theoretical coordinates ${\left[u_{t},v_{t}\right]}^{T}$ and the system aberration parameter vector k from Section 3.1, and the output layer outputs the sub-pixel-level corrected coordinate positions ${\left[u_{c},v_{c}\right]}^{T}$ corresponding to the grid. The network structure is shown in Figure 5.

At this stage, the forward propagation process of the network can be formulated as

(12) $$\begin{array}{l} x={\left[u_{t},v_{t},k_{1},k_{2},k_{3},p_{1},p_{2}\right]}^{T}\\ z^{\left(1\right)}=W^{\left(1\right)}x^{T}+b^{\left(1\right)},\;a^{\left(1\right)}=\mathrm{ReLU}(z^{\left(1\right)})\\ z^{\left(2\right)}=W^{\left(2\right)}a^{\left(1\right)}+b^{\left(2\right)},\;a^{\left(2\right)}=\mathrm{ReLU}(z^{\left(2\right)})\\ z^{\left(3\right)}=W^{\left(3\right)}a^{\left(2\right)}+b^{\left(3\right)},\;a^{\left(3\right)}=\mathrm{ReLU}(z^{\left(3\right)})\\ {\left[u_{c},v_{c}\right]}^{T}=W^{\left(4\right)}a^{\left(3\right)}+b^{\left(4\right)} \end{array}$$

where $W^{\left(l\right)}$ and $b^{\left(l\right)}$ denote the weight matrix and the *l*-th bias vector, respectively, and $z^{\left(l\right)}$ and $a^{\left(l\right)}$ correspond to the linear transformation output and activation output of the *l*-th layer, respectively.

The definition and calibration method of the aberration parameter vector $k={\left[k_{1},k_{2},k_{3},p_{1},p_{2}\right]}^{T}$ are described in detail in Section 3.1. The hidden layers adopt the ReLU activation function, and the output layer adopts the linear activation function Softmax to ensure the continuity of the output coordinates. To optimize the network parameters, the mean squared error between the theoretical coordinates, the corrected coordinates, and the coordinate ${\left[u_{d},v_{d}\right]}^{T}$ mapped by the aberration field model is defined as the loss function as follows:

(13) $$L=\frac{1}{N}\sum_{i=1}^{N}\left[{\left(u_{d}-u_{t}\right)}^{2}+{\left(v_{d}-v_{t}\right)}^{2}\right]$$

The Adam optimizer is employed to minimize this loss function. A validation set is reserved during training, and an early stopping strategy is adopted to prevent overfitting. After training is completed, the network parameters are saved for subsequent real-time inference.

It should be noted that the correction target of the proposed method is the star simulator itself, rather than the combined system of the star simulator and a specific star tracker. The training data are derived from the direct measurements of the output spots of the star simulator using a high-precision theodolite. Therefore, the accuracy of the corrected star simulator is determined relative to the theodolite as an absolute reference. Under the premise that the working conditions of the star simulator remain stable, the correction model can be effective for a long time without repeated training. The accuracy of the simulated star map output by the star simulator remains unchanged regardless of the type of star tracker replaced, which provides a unified, traceable, and high-precision benchmark for the ground calibration of star trackers.

### 3.3. Real-Time Inference and Star Map Correction

During the real-time inference and star map correction stage, the task is to apply the trained MLP network to any input ideal star map, directly output the sub-pixel-level corrected coordinates, and drive the display device to generate a high-precision simulated star map.

In the inference stage, the trained MLP network establishes an end-to-end mapping from ideal coordinates to corrected coordinates, which can quickly predict the corresponding corrected coordinates for any input ideal star point coordinates (including star points not involved in training), thus avoiding the complex iterative calculation and partition correction processes in traditional methods.

For any input ideal star point coordinates, combined with the system aberration parameters calibrated in Section 3.1, the corresponding corrected coordinates are directly output through forward propagation, which can be expressed as follows:

(14) $${\left[u_{c},v_{c}\right]}^{T}=f_{MLP}\left({\left[u_{t},v_{t},k_{1},k_{2},k_{3},p_{1},p_{2}\right]}^{T}\right)$$

This learning-based mapping relationship endows the network with the capability of real-time prediction over the entire field of view. This process does not require retraining or local interpolation, thereby achieving real-time correction across the full field of view. The corrected star point coordinates directly drive the star map display device; combined with the super-resolution display technology described in Section 2.2, high-precision localization of the energy center of star points is achieved, ultimately generating simulated star maps that satisfy the accuracy requirements.

### 3.4. Algorithm Performance Verification

The algorithm performance verification stage seeks to comprehensively validate the effectiveness, stability, and generalization capability of the proposed method through quantitative evaluation so as to ensure that all stages of the workflow cooperate to achieve the expected accuracy.

**Figure 6:**
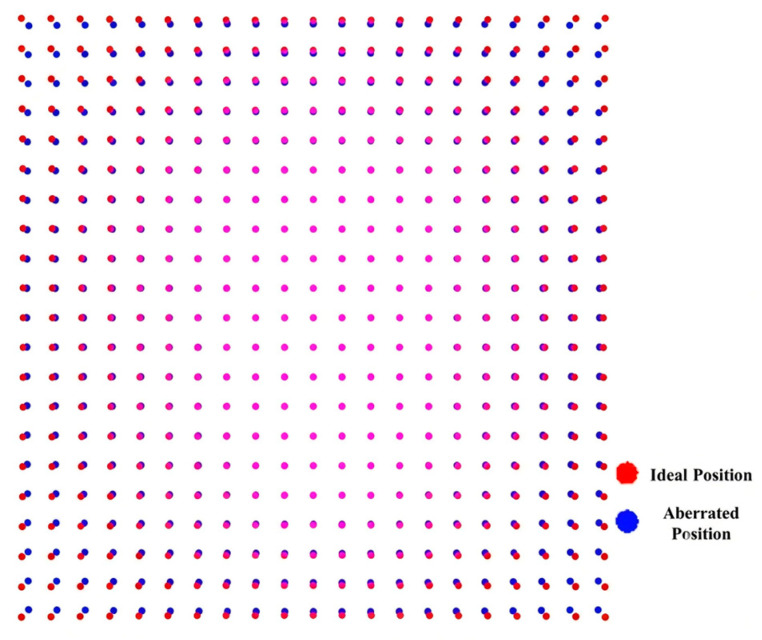
Positional distributions of calibrated grids before and after aberration field mapping.

The training set adopts a 21 × 21 uniformly distributed calibration grid. A radial distortion of 2% is introduced to simulate the aberration field of the optical system. The position distributions of the calibration grid before and after mapping by the aberration field are shown in Figure 6.

**Figure 7:**
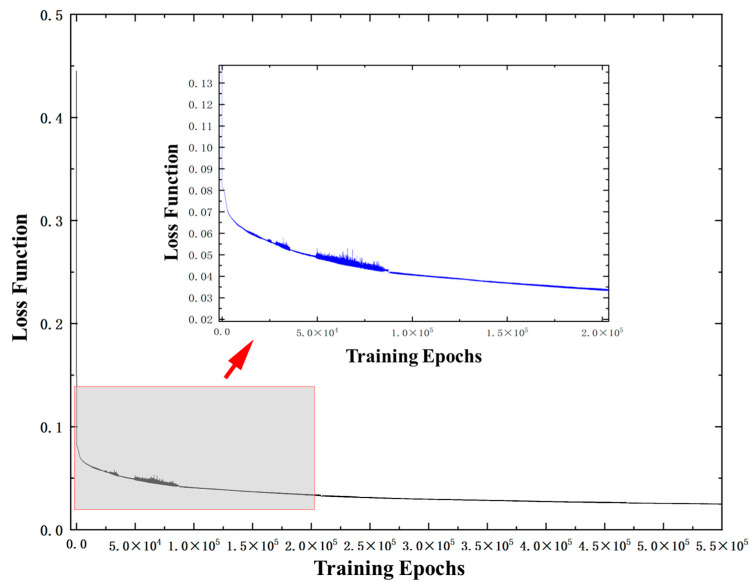
Evolution curve of the loss function for the MLP network.

After setting the network hyperparameters, an end-to-end optimization is performed using a sample-by-sample training strategy. As the training iterations proceed, the loss function of the MLP network exhibits a stable convergence trend, and the training loss curve is shown in Figure 7.

For a clearer observation of the convergence details in the later training stage, a locally magnified view of the loss function during iterations is included in Figure 7. The magnified view demonstrates that after an initial rapid drop, the model enters a stable and fine convergence phase. The loss fluctuates within a small range and decreases gradually and continuously, with no obvious oscillation or overfitting observed, which validates the stability and reliability of the model training process.

Building upon this framework, a comparative assessment of pixel positional accuracy was conducted for the algorithm both pre- and post-correction, leveraging two complementary validation paradigms: intra-training-set validation and cross-dataset validation. This dual-validation strategy enabled a comprehensive quantification of the algorithm’s overall performance metrics.

#### 3.4.1. Intra-Training-Set Validation

**Figure 8:**
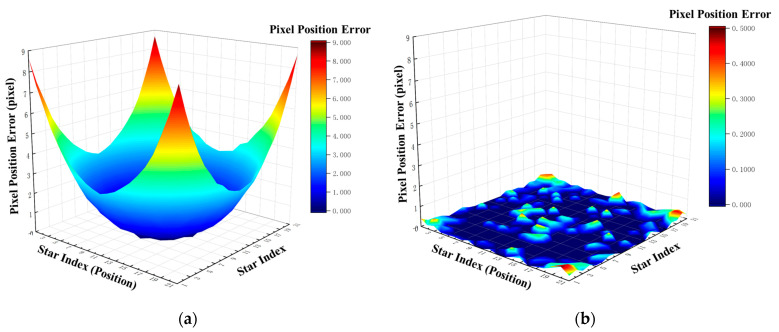
Star spot coordinate error comparison (pre- vs. post-correction) in intra-training-set validation: (**a**) pixel positional errors before correction; (**b**) pixel positional errors after correction.

The theoretical coordinates of each star point in the training set were input into the trained MLP network to obtain the corresponding corrected coordinates. Subsequently, these corrected coordinates were mapped through the system aberration field model to yield the final imaging positions, whose errors were calculated by comparison with the theoretical positions. A comparison of the pixel position errors of the star point coordinates before and after correction is illustrated in Figure 8.

In Figure 8, the horizontal axis “star index” represents the sequential number of the 21 × 21 calibration grid points, totaling 441 points. The left and right subplots adopt different scales to clearly display the sub-pixel-level error distribution.

The results show that the maximum error is 8.67 pixels with a mean value of 2.22 pixels without correction; the maximum error is reduced to 0.49 pixels with a mean value of 0.07 pixels after correction, achieving sub-pixel-level accuracy. This validates the effectiveness of the first three stages of the proposed framework (calibration, modeling, and training).

#### 3.4.2. Cross-Dataset Validation

**Figure 9:**
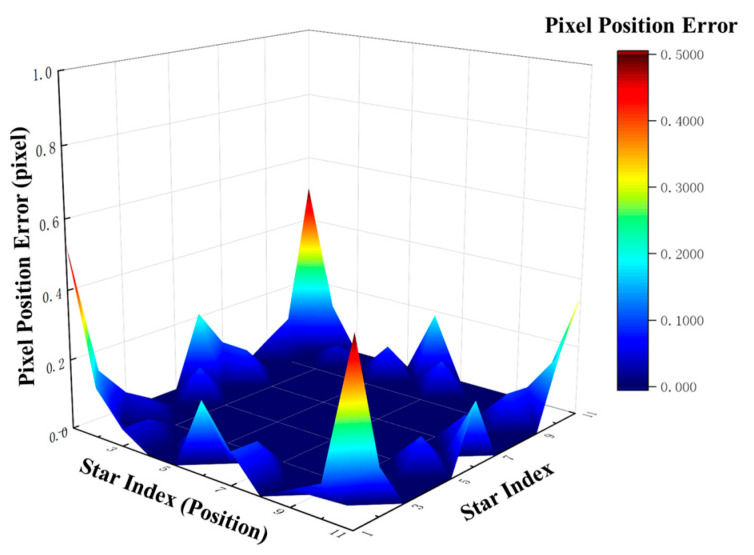
Post-correction positional error distribution of star spot coordinates in cross-dataset validation.

To further evaluate the generalization capability of the model, an independent 11 × 11 test grid was adopted for validation. The theoretical coordinates of the test set were input into the trained MLP network, and the imaging positions were obtained after being mapped through the system aberration field. The distribution of deviations between these imaging positions and the theoretical positions is illustrated in Figure 9. The maximum position deviation is 0.54 pixels with a mean value of 0.04 pixels, and the errors remain concentrated at the sub-pixel level.

The results indicate that the model maintains stable correction performance on unseen test data, verifying that the entire technical pipeline exhibits superior generalization capability and laying a solid foundation for practical engineering applications.

## 4. Experiments and Analysis

To verify the effectiveness of the MLP-based star map correction method proposed in this paper, the long exit-pupil distance dynamic spatial small target simulation system developed in Ref. [22] was employed as the core display device, and an experimental platform was constructed using a theodolite with equivalent precision. The accuracy correction experiment was first conducted using the proposed method, followed by the performance verification experiment.

For a direct and fair comparison with the results reported in Ref. [22] to quantitatively evaluate the improvement of the proposed method over previous work, two real star images used in that study were selected as test samples, and the same core evaluation metrics, namely single-star position error and inter-star angular distance error, were adopted.

The traditional comparative method [22] is a grid calibration-based geometric correction approach. A 10 × 10 uniform grid is set across the full field of view as calibration points, and the actual position of each grid point is measured by a high-precision theodolite to establish the correspondence between ideal and measured coordinates. On this basis, polynomial fitting (usually second- or third-order polynomials) is applied to model the distortion distribution over the entire field of view, and the corrected coordinates of arbitrary star points are computed accordingly.

This method improves the positioning accuracy of the star simulator to a certain extent. However, restricted by the sparsity of the calibration grid, its capability to describe local distortion is limited, and the order selection of the fitting model lacks unified theoretical guidance. It is employed as the baseline method for comparison in this work.

It should be noted that the proposed method is universal and independent of specific hardware configurations. The same experimental platform as in previous research is utilized here only to achieve a direct and fair comparison with [22], so as to reliably quantify the advancement of the proposed method over prior work. For other types of star simulator systems, the proposed method can be applied by re-conducting system calibration and network training only.

**Figure 10:**
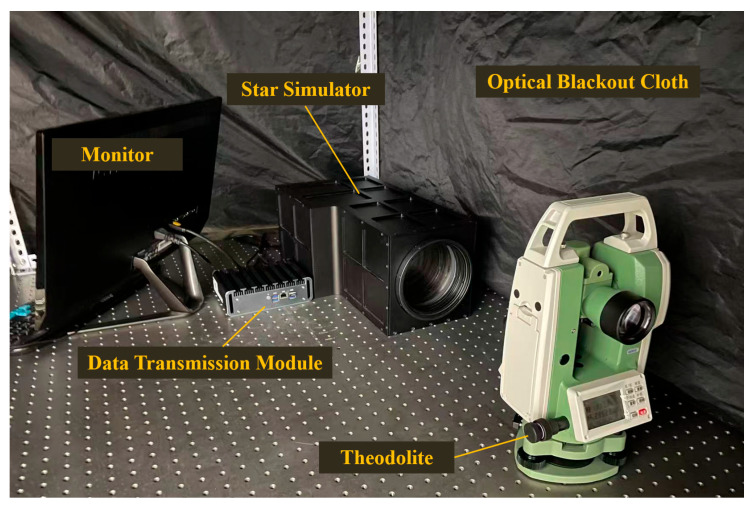
Experimental platform.

The experimental platform is illustrated in Figure 10.

**Figure 11:**
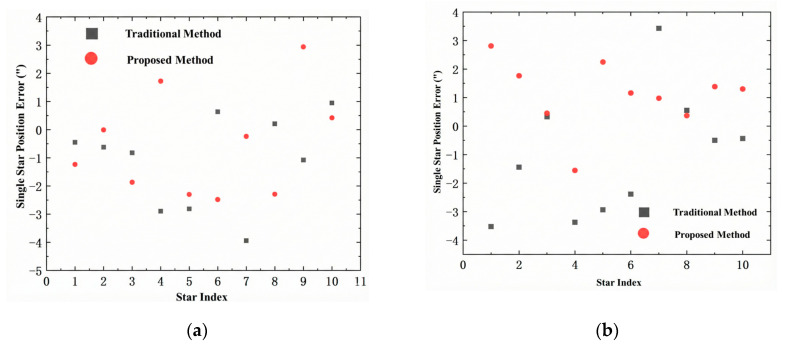
Comparison of star position errors for Star Map 1 and Star Map 2: (**a**) error scatter distribution of Star Map 1; (**b**) error scatter distribution of Star Map 2.

The comparison results of the single-star position error between the proposed method and the traditional method described in [22] are illustrated in Figure 11. The traditional method adopted in [22] is based on a 10 × 10 calibration grid, where the actual positions of each grid point are measured point by point using a high-precision theodolite. After obtaining the calibration data, the field of view is divided into multiple quadrants, and high-order polynomial fitting is performed separately in each quadrant to establish the mapping relationship from ideal coordinates to the measured coordinates. Although this method improves the accuracy compared with the uncalibrated system, it suffers from problems such as discontinuity at partition boundaries and limited correction ability in the edge regions with high distortion.

The star position error is closely related to the field of view position. The difference in aberration characteristics in different field regions leads to a strong field-dependent error distribution, rather than a regular variation with the star index. In practical engineering applications, the accuracy evaluation of star simulators usually takes the maximum error within the full field of view as the core indicator, since the maximum error represents the worst-case performance of the system and directly determines its limiting performance. This paper adopts this evaluation criterion and focuses on the improvement of the maximum error.

A comparison of the maximum, minimum, and average star point position errors derived from Star Map 1 and Star Map 2 between the proposed method and the traditional method is presented in Table 1.

Let the error of the traditional method be *a* and that of the proposed method be *b*; then, the performance improvement is $\frac{\left|b|\;-\;|a\right|}{\left|a\right|}100\%$. Table 1 demonstrates that the proposed method yields comprehensive accuracy enhancements in Star Map 1. The maximum error is reduced from 3.94″ to 2.94″, corresponding to a 25.4% accuracy improvement; the minimum error is optimized to −0.01″, nearing the theoretical zero; and the average error decreases from 1.08″ to 0.53″, translating to a 50.9% accuracy gain, with a marked narrowing of the overall error distribution. In Star Map 2, the maximum error is reduced by 20.4% to 2.81″, the minimum error remains stable, and the average error is maintained at approximately 1.0″, thus showcasing robust overall error control capability.

**Figure 12:**
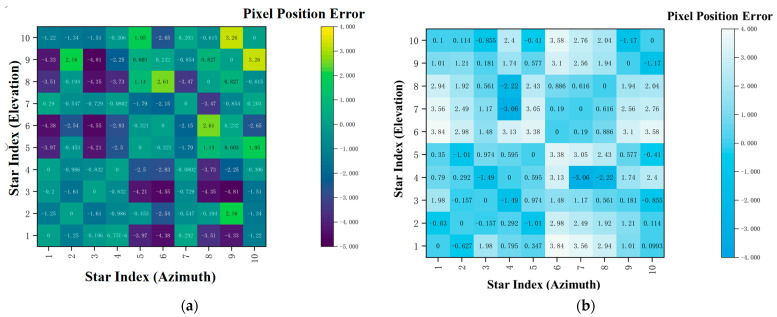
Color-coded maps of inter-star angular distance errors: (**a**) color-coded map of inter-star angular distance errors for Star Map 1; (**b**) color-coded map of inter-star angular distance errors for Star Map 2.

Integrating the results of the two star maps, the method proposed in this paper outperforms the conventional method in all three core metrics. Specifically, the maximum error is reduced by an average of 22.9%, thereby significantly enhancing the reliability of the system; the minimum error is consistently maintained below 0.3″, demonstrating superior local accuracy retention capability; and the average error exhibits an overall decreasing trend. Experimental results verify that the proposed MLP correction method can effectively suppress extreme deviations, preserve local positioning accuracy, and constrain the overall system error, thereby comprehensively enhancing the integrated accuracy and stability of star spot localization. At this point, the color-coded maps of the inter-star angular distance error distribution for the star maps are shown in Figure 12. The color in the figure represents the magnitude of the angular distance error between each star pair, and the color bar on the right provides the correspondence between error values and colors.

Since the minimum inter-star angular distance error of the star maps is 0, the comparison of the maximum and average inter-star angular distance errors between the proposed method and the traditional method for Star Map 1 and Star Map 2, derived from Figure 12, is presented in Table 2.

It should be noted that, constrained by the requirement to maintain consistent experimental conditions with Reference [22], only two real star images are employed for experimental validation in this paper, leading to a relatively limited sample size.

Based on the comparison results of inter-star angular distance errors presented in the table, the method proposed in this paper exhibits remarkable advantages in suppressing the maximum error. For Star Map 1, the maximum inter-star angular distance error is reduced from 6.41″ to 4.81″, representing a precision improvement of 24.9%. For Star Map 2, this error is sharply decreased from 7.69″ to 3.84″, with the precision improvement reaching as high as 50.1%. Meanwhile, the average inter-star angular distance errors of the two star maps remained stable, staying at approximately 1.3″ and 1.0″, respectively. Overall, the maximum inter-star angular distance errors were reduced by an average of 37.5%, with the distribution of the average errors remaining steady.

## 5. Conclusions

This paper addresses the correction of star-point positioning errors in dynamic star simulators, proposing and experimentally validating a super-resolution star map correction method based on an MLP. By establishing a complete technical pipeline of “system calibration–aberration field modeling–network-based correction”, a data-driven end-to-end correction framework was developed, enabling the unified modeling and compensation of multiple error types, including optical aberrations, alignment deviations, and device discreteness. At the theoretical level, the limitations of traditional geometric correction were elaborated, and a super-resolution correction mechanism based on energy regulation was introduced, thereby laying a solid theoretical foundation for the sub-pixel-level localization of star spots. At the algorithmic level, a full-process scheme encompassing aberration field calibration, MLP network training, and real-time inference was designed. By learning the aberration distribution characteristics contained in the calibration data, the MLP network acquires the capability to predict the coordinates of arbitrary ideal star points, thereby achieving high-accuracy correction of star point positions over the entire field of view.

Building upon the theoretical and algorithmic foundations, systematic performance tests were conducted on the proposed model in this study. The experimental results demonstrate that, on the 21 × 21 training set grid, the maximum star spot positioning error was reduced from 8.67 pixels to 0.49 pixels, while the mean error was significantly decreased from 2.22 pixels to 0.07 pixels, thus achieving sub-pixel-level positioning accuracy. Furthermore, an independent 11 × 11 test set was employed to verify the generalization capability of the model. The results indicate a maximum positional deviation of 0.54 pixels and a mean deviation of 0.04 pixels, with all errors remaining confined within the sub-pixel range. The star point coordinates in this independent test set were not involved in network training. The output of the corrected coordinates by the model is exactly the prediction based on the learned aberration distribution law. The results demonstrate that the proposed method possesses favorable generalization performance and stability.

In practical star map experiments, the proposed method achieved comprehensive and significant performance improvements on two captured star maps: the average reduction rate of the maximum star-point positioning errors reached 22.9%, with the mean error exhibiting a distinct downward trend; the average reduction rate of the maximum inter-star angular distance errors reached 37.5%, while the mean angular distance error remained stable. Experimental results show that the proposed method can effectively suppress extreme angular distance deviations without compromising the stability of the overall error distribution. Consequently, it achieves superior comprehensive performance in preserving the relative positional relationship between star points and significantly outperforms traditional methods in terms of star positioning accuracy, star map structure consistency, and system adaptability.

For practical implementation, the system developed in this study exhibits clear potential for engineering deployment. Its modular and compact optical architecture can be readily integrated into the existing calibration workflow of star simulators and directly serve as the reference light source for ground-based testing of star sensors. This significantly reduces the demand for mechanical alignment, lowers system complexity and cost via a software-defined solution, and enhances system reliability. The proposed method targets the calibration of the star simulator itself, and the trained correction model is independent of any specific star tracker, thus featuring strong universality and autonomy. It provides reliable technical support for high-precision star map display and ground calibration of star sensors, demonstrating distinct engineering application value. In future work, the proposed scheme can be further developed into a portable field calibration instrument to meet the high-precision testing requirements in spacecraft navigation, astronomical observation, and other related fields.

In summary, the MLP-based correction network developed in this study effectively integrates the coupled effects of multiple factors, including optical aberrations, alignment deviations, and device discreteness, through an end-to-end data-driven mechanism. It exhibits significant advantages in enhancing positioning accuracy, maintaining structural consistency, and improving generalization capability. On the basis of the above work, in future research, we will first acquire more star images with diverse distribution patterns, magnitude ranges, and field-of-view coverages so as to carry out more comprehensive evaluations and validations of the proposed method and further verify its performance boundaries and generalization capability.

## Figures and Tables

**Table 1:** Comparison of maximum, minimum, and average star point position errors with the traditional method.

Star Map Index	Performance Metrics	Traditional Method [22]	Proposed Method	Performance Improvement
Star Map 1	Maximum Star Point Position Error (″)	−3.94	2.94	Accuracy is improved by 25.4%
	Minimum Star Point Position Error (″)	0.21	−0.01	Close to the theoretical accuracy
	Average Star Point Position Error (″)	−1.08	−0.53	Accuracy is improved by 50.9%
Star Map 2	Maximum Star Point Position Error (″)	−3.53	2.81	Accuracy is improved by 20.4%
	Minimum Star Point Position Error (″)	0.32	0.36	Maintain stability
	Average Star Point Position Error (″)	−1.03	1.09	Maintain stability

**Table 2:** Comparison of maximum and average inter-star angular distance errors with the traditional method.

Star Map Index	Performance Metrics	Traditional Method [22]	Proposed Method	Performance Improvement
Star Map 1	Maximum inter-star angular distance error (″)	−6.41	−4.81	Accuracy is improved by 24.9%
	Average inter-star angular distance error (″)	−1.26	−1.56	Maintain stability
Star Map 2	Maximum inter-star angular distance error (″)	−7.69	3.84	Accuracy is improved by 50.1%
	Average inter-star angular distance error (″)	−1.26	1.04	Maintain stability

## Data Availability

The data presented in this study are available upon request from the corresponding author.

## Abbreviations

MLP	Multi-layer perceptron
FOV	Field-of-view
PSF	Point spread function
MSE	Mean squared error
